# Violence and trauma towards children and adolescents in the July mass uprising (2024) in Bangladesh: a socio-demographic analysis of 89 deaths 

**Published:** 2025-07

**Authors:** S.M Yasir Arafat, Md. Sabbir Sheikh, Mohammad Sorowar Hossain

**Affiliations:** ^*a*^ Biomedical Research Foundation, Dhaka, Bangladesh.; ^*b*^ Department of Psychology, University of Rajshahi, Rajshahi-6205, Bangladesh.

**Keywords:** Mass Uprising, Children, Violence, Bangladesh, Quota Protest, July Massacre, Student Killing

## Abstract

**Background:**

Bangladesh has experienced a mass uprising that resulted in deaths, injuries, and trauma, ended with the ousting of an autocratic government on 05 August 2024. People from all spheres of life have experienced violence and trauma, including children and adolescents. We aimed to report the socio-demography of 89 children killed in the mass uprising in Bangladesh in July-August 2024.

**Methods:**

We extracted the data of this report from a widely circulated Bangla national daily newspa-per. We extracted name, age, occupation, place of death, and district of killing.

**Results:**

The mean (SD) age of the deceased was 15.2 (±2.6) years, ranging from 4-17 years. Among the 89 deaths, there were bullet wounds in 79 children, 9 died by burn, and one died by splinter; 56 deaths happened during 18 July- 4 August, and 31 deaths happened after 5 August, 2024; 58 deaths happened in Dhaka, and 31 deaths happened in districts outside Dhaka. Among the adolescents, 42 were students, and 29 were involved in child labor. Deaths happened in 16 districts in Bangladesh.

**Conclusions:**

This analysis revealed that about 90% of the adolescents were killed by bullets, indicating the spectrum of armed conflict. Dhaka was the center of violence that resulted in the killing of adolescents. Local, regional, and international human rights agencies should ensure initiatives to prevent such killings of children during any mass protest elsewhere in the world.

## Introduction

Bangladesh witnessed a violent mass uprising led by university students protesting the reinstatement of a controversial quota system for civil service positions in early July 2024. It started after the High Court’s decision to reinstate the quota system, which had been abolished in 2018 following extensive protests, and triggered widespread unrest among university students, primarily.^1,2^ This movement, known as the anti-quota movement, became one of the most violent episodes in Bangladesh’s history since the Liberation War of 1971, ultimately contributing to the ousting of the The term "quota" typically refers to a system of political or social allocation used to ensure representation for various communities, such as ethnic, religious, or gender (male and female), within a government or organization. In 1972, the Bangladesh government introduced a quota system that reserved a percentage of government jobs for the descendants of those who fought in the 1971 Liberation War. Under this system, 44% of government jobs were based on merit, while the remaining 56% were reserved for specific groups: 30% for descendants of freedom fighters, 10% for women, 10% for candidates from underdeveloped districts, 5% for ethnic minorities, and 1% for individuals with disabilities. This quota system was abolished in October 2018 after widespread protests.^2^ The current anti-quota movement is focused on abolishing the 30% quota for descendants of freedom fighters, while still supporting the reservation of jobs for ethnic minorities and people with disabilities.

**Extent of violence and injury**

The United Nations (UN) reported that at least 1400 people were killed, more than 11,700 people were arrested and detained.^3^ The casualties included innocent civilians, children, protesters, bystanders, journalists, and members of the security forces. The majority of these deaths were attributed to the actions of security forces, including the Police, Rapid Action Battalion (RAB), Border Guard Bangladesh (BGB), and affiliated groups of the ruling Awami League, such as the youth wing (Jubo League), volunteers wing (Swechchhasebak League), and student wing (Chhatra League). These groups were reported to be armed with illegal firearms and crude weapons, contributing to the violence that has since been labeled the ‘July Massacre.’ Along with deaths, it causes eye injury to more than 1300 persons, and 630 persons required eye surgery.^4^

**Injury and violence towards children**

Under the Children Act of 2013, a child is defined as a person below the age of 18 years. According to Bangladesh’s National Child Policy of 2011, "Children shall include all individuals under 18 years," and "Children below the age of 14 to 18 years are to be considered as adolescents".^5^ The latest report of the UN revealed that among the 1400 deaths, approximately 12-13% were children.^3^ A tragic example of this is the death a four-year-old preschooler who was shot and killed on July 19, at around 1:30 p.m., while standing on the eighth-floor balcony of his house. Another deceased was a six-year-old female student who was shot dead by a helicopter while playing on the roof of her house. Over time, additional reports have provided more details on the children killed during the protests. Initially, the reported number was 70. However, later, one report mentioned that 132 children and adolescents were killed during the protest.^6^ However, we did not find the detailed socio-demography of them. However, a holistic socio-demographic analysis of the deaths of children has not been conducted. Therefore, our aim was to report on the socio-demography of 89 children killed in this mass uprising in Bangladesh that is reported in the national newspaper.

## Methods 

Medical record-keeping in Bangladesh is severely inadequate compared to developed countries, and this shortcoming was starkly exposed during the conflict. The entire healthcare system in the city almost crumbled under the pressure, with some hospital records reportedly seized by police in an attempt to cover up the identities of the deceased, as reported by newspapers.^3,7^ During the clashes, most of the injured and dead individuals were brought to the hospitals by protesters and common people. In some cases, the police also brought them to the hospital, often without knowing their identities, ages, or occupations. Despite these challenges, journalists meticulously tracked down many victims' families, gathering vital information and hospital records to uncover the truth.^8^ We extracted the data of this article from a widely circulated Bangla national daily newspaper, where the socio-demography of 89 children was available, despite the total number of deaths is higher.^9^ We extracted name, age, occupation, place of death, and district of killing. We utilized Microsoft Excel version 10 for Windows and presented data descriptively. We collected the data from daily newspaper. Therefore, no formal ethical approval was sought. We did not present the proposal to any institutional review board. 

## Results

**Figure 1:**
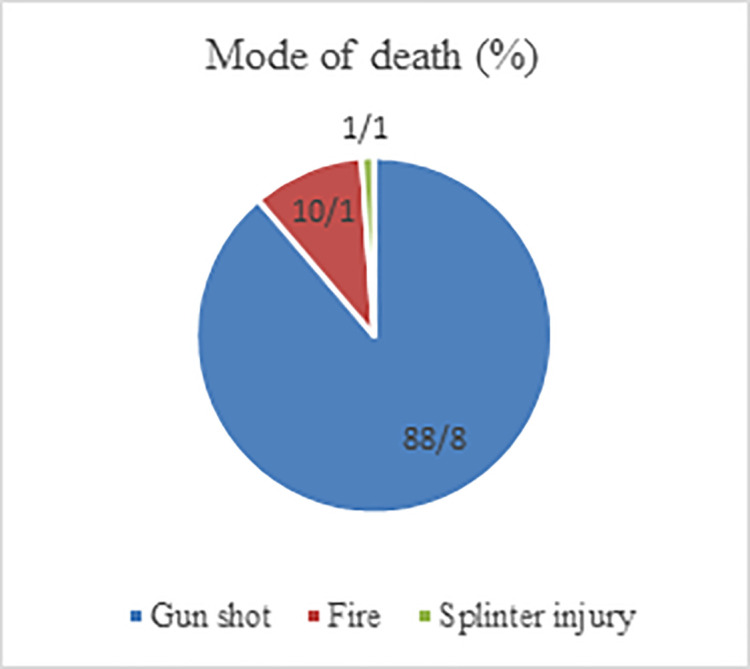
Mode of death of 89 child and adolescents

We found the socio-demographic details of 89 children and adolescents from a national daily newspaper. The mean (±SD) age of the deceased was 15.2 (±2.6) years, ranging from 4 to 17 years. Among the 89 deaths, 6 were of children aged 10 years or younger, 26 were in the 11-15 years age group, and 56 were aged 16 or 17 years (Table 1). About 98% of deceased were males. Among the deceased, 79 (89%) were killed by bullets, 9 died from burns, and 1 death resulted from a splinter injury (Figure 1). Fifty-six deaths occurred between July 18 and August 4, while 33 deaths occurred between August 5 (the day when the regime fall) and August 11. Fifty-eight deaths took place in Dhaka, and 31 occurred in districts outside the capital. Among the deceased, 42 were students, and 29 were involved in child labor. The deaths were recorded across 16 districts in Bangladesh.

**Table 1:** Profile of 89 children’s deaths during mass up rise in Bangladesh

Variable	n	%
**Age in years**		
4-10	6	6.7
11-15	26	29.2
16-17	56	62.9
**Sex**		
Male	87	97.8
Female	2	2.2
**Occupation**		
Factory worker	10	11.2
Hawker	5	5.6
Shopkeeper	5	5.6
Rickshaw driver	3	3.4
Others	2	2.2
Workshop worker	3	3.4
Student	42	47.2
Not Known	19	21.3
**District**		
Dhaka	58	65.2
Bogura	1	1.1
Chittagong	1	1.1
Cumilla	5	5.6
Gaziour	3	3.4
Habiganj	3	3.4
Kustia	2	2.2
Magura	1	1.1
Narayanganj	6	6.7
Narsingdi	3	3.4
Natore	1	1.1
Noakhali	1	1.1
Pabna	1	1.1
Satkhira	1	1.1
Tangail	1	1.1
Thakurgaon	1	1.1
Total	89	100.0

## Discussion

**Major findings of the study**

The socio-demographic analysis of 89 children and adolescents killed in the July Mass Uprising revealed that children were killed from 4 to 17 years, about 98 were males, about nine out of ten children were killed by bullets, about two-third of deaths were happened in Dhaka, and more than half of the deaths happened among non-students. It indicates the extent of the protest, the presence of all walks of life (from kids to older adults) in the protest. The quota movement was initially started by the university students in early July, 2024. Later, people from all spheres of life joined it to support the demand and to protest the violence. At the same time, it also indicates the violence and crackdown that happened during the period, with massive use of firearms on the protesters, as about 90% of the deaths among children were due to gunshot. It resulted in at least 1400 deaths, 11700 arrests, curfews, block raids to detain the students, and an internet shutdown (July 18–23 and August 4–5, 2024).^3,10^ Dhaka and the nearby districts were the epicentre of protest, attributing the deaths in the areas of Dhaka and Narayanganj. 

This report highlights the number and demographics of children and adolescents who died during the anti-quota movement followed by a mass uprising against the Government in Bangladesh. It can be a historical piece validating the oppressive violence against children by an autocratic regime to stay in power against the will of the general population. Our analysis revealed 89 deaths, which may be an underreporting; the actual number could be higher.

Similar revolutions and participation of students/ children were noted in other parts of the world, especially during the Arab Spring in Egypt.^11^ However, such massive use of firearms and lethal weapons against children and adolescents was not noted. Mass protests due to a student's death were also noted in China and Nigeria.^12,13^ However, none of them were such brutal like the conditions in the July protest in Bangladesh. The July (2024) Mass Uprising was extended to such a level that some children playing on rooftops, standing on balconies, or closing windows were shot and some were killed while watching the protests.^14^ Many children and teenagers were arrested during street sweeps and home raids to refrain them joining the protest. During this time, 137 young prisoners were sent to the Child Development Center (boys) after being arrested in various cases.^15^

Some other violence on children was noted in severe extreme. On August 9, 2024, the Statista Research Department published a report stating that more than 20,000 children had been killed by March 2023 due to the Syrian Civil War, which started in 2011. Children have been victims of bombings, shootings, and other forms of violence.^16^ A UN expert reported that at least 382 children were killed during Myanmar's junta crackdown, including by airstrikes and heavy artillery.^17^ At least 23 children were killed with impunity during the brutal crackdown on youthful protests in Iran from September 20 to 30, 2022.^18^ The context of Bangladesh is different from Syria and Myanmar, as the country was not in a state of civil war.

**Implications of the study results**

Documentation of this sheer violence against children could be a historical piece validating the oppressive violence against children by an autocratic regime to stay in power against the will of the general population. Findings from this study can drive changes in policy and advocacy efforts aimed at protecting children in the protest areas. Policymakers can use these findings to advocate for specific resolutions or agreements. The results can inform humanitarian organizations about the specific needs and vulnerabilities of children in protest crises, helping to shape their emergency response plans. Insights from this study can improve operational strategies for NGOs and international organizations working in conflict zones. Additionally, the study's findings can highlight gaps in the enforcement of international laws designed to protect children, prompting necessary legal reforms. Understanding the impacts of conflict on children can also help shape long-term recovery and development strategies.

**Limitations**

Several limitations of this study should be acknowledged. The data was sourced from a Bangla daily national newspaper, and the reported number of deaths is subject to change as the situation evolves. Consequently, ongoing monitoring and careful observation are necessary.

## Conclusion

This analysis revealed that nine out of ten adolescents were killed by gunfire, highlighting the severe nature of the armed conflict. Dhaka emerged as the epicenter of violence and adolescent fatalities. Local, regional, and international human rights organizations must take action to prevent such tragedies during protests in the future.

**Data availability statement**

Data will be provided on request to the corresponding author. 

**Authors’ contributions**

Conceptualization: Mohammad Sorowar Hossain

Methodology: S. M. Yasir Arafat and Mohammad Sorowar Hossain

Project Administration: Mohammad Sorowar Hossain

Writing – Original Draft Preparation: All authors

Writing – Review & Editing: All authors

All authors have read and approved the final version of the manuscript.

## References

[1] Siddik MAB (2024). Bangladesh protests: law enforcement and public health crisis. Lancet.

[2] Faruk MO (2024). The mental health challenges of student protest in Bangladesh. Lancet Psychiatry.

[3] United Nations. Human Rights Violations and Abuses related to the Protests of July and August 2024 in Bangladesh. 2025. https://www.ohchr.org/sites/default/files/documents/countries/bangladesh/ohchr-fftb-hr-violations-bd.pdf, accessed on April 19, 2025.

[4] Islam F, Arafat SMY, Hossain MS (2025). Eye injuries in Bangladesh’s 2024 student-led mass uprising: A public health crisis unfolds. Torture.

[5] Ministry of Women and Children Affairs. National Children Policy 2011. 2011. http://childsocialprotection.gov.bd/public/upload/policy_files/202105240620National%20Children%20Policy%202011%20English.pdf, accessed on August 25, 2024.

[6] Akter N. 132 children, 11 women killed in mass uprising. Prothom Alo. https://en.prothomalo.com/bangladesh/bfrekkzrdm, accessed on April 19, 2025.

[7] Shimul T. What is known so far about the death toll in the quota movement. BBC News Bangla. 2024. https://www-bbc-com.translate.goog/bengali/articles/cek9eded8jro?_x_tr_sl=bn&_x_tr_tl=en&_x_tr_hl=en&_x_tr_pto=wapp, accessed on August 23, 2024.

[8] Ahmed R, Hassan A. Analysis of 150 deaths: 113 of the deceased young, 45 students. Prothom Alo. 2024. https://en.prothomalo.com/bangladesh/1hmcovbabm, accessed on August 27, 2024.

[9] Akter N. Student-Majority Movement: The number of children and adolescents killed has increased to 89. Prothom Alo. 2024. https://www-prothomalo-com.translate.goog/bangladesh/99w8cs0fs2?_x_tr_sl=bn&_x_tr_tl=en&_x_tr_hl=en&_x_tr_pto=wapp, accessed on August 27, 2024.

[10] Hussain R, Kabir R, Kar SK, Arafat SY (2024). Internet Shutdown with Violations of Human Rights and Freedom of Speech. Asian Journal of Public Health and Nursing.

[11] BBC News. Egyptian children tell revolution tales. https://www.bbc.com/news/education-13312429, accessed on April 19, 2025.

[12] Chin V. VOA Mandarin: Mass protest erupts in China's Shaanxi over student’s death. Voice of America. https://www.voanews.com/a/voa-mandarin-mass-protest-erupts-in-shaanxi-over-student-s-death/7931364.html, accessed on April 19, 2025.

[13] Fayokun KO (2012). Revisiting the cause of death in a student-police violent face-off. Makerere Journal of Higher Education.

[14] Reporter S. They fell before the flowers bloomed. The Samakal. 2024. https://samakal-com.translate.goog/bangladesh/article/248113/, accessed on August 23, 2024.

[15] Representative BBT. 137 juveniles arrived in 16 days after being arrested. The Samakal. 2024. https://samakal-com.translate.goog/bangladesh/article/248928/, accessed on August 23, 2024.

[16] Statista. Number of children killed in Syria from 2011 to 2023, by party responsible. 2023. https://www.statista.com/statistics/697188/child-deaths-in-syria-by-party-responsible/, accessed on August 25, 2024.

[17] Reuters. Children killed, tortured in Myanmar junta's crackdown, UN expert says. 2022. https://www.reuters.com/world/asia-pacific/children-killed-tortured-myanmar-juntas-crackdown-un-expert-says-2022-06-14/, accessed on August 25, 2024.

[18] Amnesty International. Iran: At least 23 children killed with impunity during brutal crackdown on youthful protests. 2022. https://www.amnesty.org/en/latest/news/2022/10/iran-at-least-23-children-killed-with-impunity-during-brutal-crackdown-on-youthful-protests/, accessed on August 25, 2024.

